# Less effort but equal result: Introducing the daily run-size estimation method for quantifying fish passage in fishways

**DOI:** 10.1371/journal.pone.0252183

**Published:** 2021-05-26

**Authors:** Mariana O. Côrtes, Alexandre Peressin, Alexandre L. Godinho

**Affiliations:** Fish Passage Center, Zoology Department, Federal University of Minas Gerais, Belo Horizonte, MG, Brazil; Swedish University of Agricultural Science, SWEDEN

## Abstract

Determining the number of fish that use a fishway is essential to fisheries management but counting all fish can be impracticable due to labor and cost. We present the daily run-size estimation (DARSE) method, which uses systematic sampling to estimate the number of fish per species that pass through a fishway daily (daily run size, D). The DARSE method makes it possible to determine the minimum fraction of each hour (or hourly samples) of the day necessary to estimate D with known accuracy. We apply DARSE to each of the seven most abundant fish species (other species grouped under ‘Others’) recorded in video images taken during 46 days of one year at the Igarapava Fish Ladder, Brazil. Accuracy in estimating D was influenced by the fraction of the hour sampled and the temporal pattern of fish passage through the fishway. For species with a more uniform temporal pattern of passage, the DARSE method reduced the time spent on sampling by up to 96%, depending on the accuracy used to estimate D. Some of these species required counts of fish that pass in a fraction of an hour for all hours of the day while counts for other species can be done every 2 hours or, more rarely, every 3 hours. For species with a more aggregated temporal pattern of passage, it was possible to estimate D by sampling a fraction of an hour but with reduced accuracy in the estimation of D and little reduction in sampling time.

## Introduction

Blocking migration is one of the main causes of reduced abundance of migratory river fish whereas fishways have been one of the most used strategies for mitigating this impact [1–3]. Estimating the number of fish that past a fishway (run size) is important for evaluating the contribution of the fishway to mitigating the impact, as well as for addressing various fisheries conservation and management issues [4–7]. Estimating run size has been done as fish pass a reference point, like a viewing station [7], where fish can be seen and counted.

Ideally, all fish should be counted to estimate run size, which is possible when run size does not surpass the counting capability of the staff. If the number of fish exceeds counting capability, sampling can be used to estimate run size. However, sampling to efficiently estimate run size is complicated by annual variation in timing, duration, daily pattern, and run size [4]. Therefore, various sampling designs to estimate run size have been evaluated [4, 6, 8, 9].

Visual counts to estimate run size was done in early studies using simple random or systematic samplings with a fraction of an hour (e.g., 5 or 10 min) as a sample unit (e.g., [10, 11]). Subsequently, Jessop & Harvie [4], Davies et al. [6] and McCormick et al. [9] applied retrospective sampling—the use of historical data to set future sampling goals [12]—to compare estimates of run size among sampling designs and determine the required sample size for each. These three retrospective sampling studies knew the true run size, so accuracy and precision of the estimates could be investigated.

In their pioneer retrospective sampling study, Jessop & Harvie [4] compared run size estimates among five sampling designs based on a 15-min sample unit. They used the same two sampling designs (simple random and systematic) used in the early studies of Becker [10] and Rideout et al. [11] but included stratification by day or hour. Jessop & Harvie [4] concluded that: (i) sample size varies between sampling designs and desired accuracy; (ii) stratification reduces sample size; (iii) shorter sample units taken more frequently provide the best results for a given sampling effort; and (iv) systematic sampling may reduce sampling bias and provide more precise estimates of run size because it is spread more evenly over the population. The retrospective sampling study of Davies et al. [6] evaluated two sampling designs (simple random and simple random stratified by day), also for a sample unit of 15-min. They concluded, like one of the various conclusions of Jessop & Harvie [4], that the sample size required for stratified design is smaller than that required for non-stratified design.

The studies of Jessop & Harvie [4] and Davies et al. [6] estimated run size for a single species using a fraction of an hour as the sample unit. Differently, the retrospective sampling study of McCormick et al. [9] estimated run size for multiple species/run-types and used day as the primary sample unit and hour (or group of hours) as the secondary sample unit. McCormick et al. [9] evaluated five sampling designs and concluded that one- and two-stage stratified designs were more accurate than simple random sampling designs for most species/run-types.

The sample units of the aforementioned studies were a fixed fraction of an hour, such as the 15-min or 20-min used by Jessop & Harvie [4], Davies et al. [6] and Nelson [8], or the hour, group of hours or day as in McCormick et al. [9]. In the present study, we introduce the daily run-size estimation (DARSE) method, which determines the minimum fraction of each hour of the day or hourly samples necessary to estimate daily run size with known accuracy. The DARSE method uses systematic sampling stratified by hour to estimate daily run size by linear regression. We developed the DARSE method using retrospective sampling and applied it to various species in a fishway of a large river in Brazil.

## Methodology

**Fig 1:**
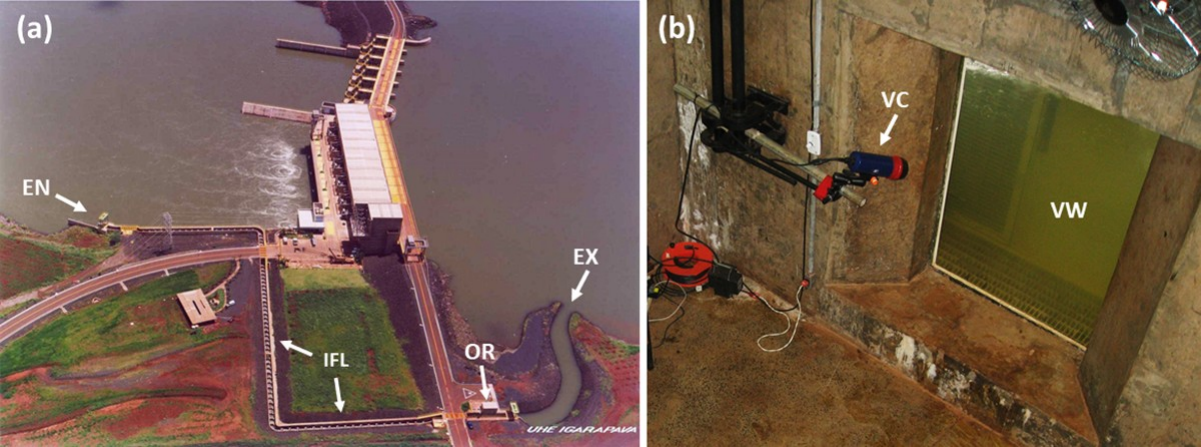
Igarapava Fish Ladder (a) and its viewing window (b). The entrance (EN), observation room (OR) and exit (EX) of the Igarapava Fish Ladder (IFL) (panel a) and the viewing window (VW) of the observation room with the video camera (VC) set inside a protective housing (panel b).

We used fish passage data from the Igarapava Hydropower Dam (19^o^ 59’ 36” S, 47^o^ 45’ 32” W) located in the Grande River (upper Paraná River basin) on the border between the states of Minas Gerais and São Paulo, Brazil. It is the eighth of 12 hydropower dams constructed along the 1,360 km extension of the river. The Igarapava Fish Ladder (IFL), in operation since 1999, is a vertical slot fishway that is 17 m tall with 6% declivity. It possesses 87 3x3-m tanks interconnected by a 0.40-m wide vertical slot [13]. The total extension of the IFL is 446 m. It has an observation room located 113 m from the fishway exit with a 1-m wide and 1.5-m tall window for viewing fish (Fig 1).

Environmental data of reservoir water level, dam discharge and rainfall was provided by the concessionaire of the dam. Water level in the IFL was determined from a gauge at the viewing window and water temperature was measured at every hour using an Onset datalogger. These environmental variables showed the following ranges for the days we counted fish: water level in the reservoir (512.0–512.4 m.a.s.l), turbine (590–1,195 m^3^.s^-1^) and spillway (0 m^3^.s^-1^) discharges, water level in the IFL (1.5–2.0 m) and water temperature (16.9–30.4 ^o^C) in the IFL. Rainfall occurred on 13 days and ranged 1.5–86.0 mm.

At least 24 species of fish, including eight migratory fishes, are knowing to use the IFL [14, 15]. Passage of fish through the IFL occurred throughout the year, but most fish taxa passed more during the wet season [14]. Regarding diel variation, the passage of some taxa peaked during the day and others at night. Moon phase had a medium or large effect size on the number of fish that passed the IFL for almost all taxa [14].

The passage of fish in front of the window was continuously recorded in time-lapse from June 1 2003 to May 31 2004 with a Sanyo VCC-4594 camera, a Sony SVT-LC300 video cassette recorder and an infrared lamp for night lighting [14]. Recording did not occur on 38 days when the IFL was not in operation. The video images of 46 days drawn at random were examined by a technician. We drew one day per moon phase for each lunar cycle because of the important effect size that it had on fish passage at the IFL. For every minute of the 46 days, the technician counted and identified all fish that passed through the display going upstream. Fish that fell-back were not counted. Water turbidity on the selected days was low enough to allow species identification of all counted fish, except for the two species of *Cichla* that occur in the region. All fish counting data used in the analyses are provided in S1 Dataset.

For each species, day and hour, we established the number of fish (F) that passed through the display for the sample unit (SU) from 5 to 60 min in 5-min increments. Thus, for the SU of 5 min (SU_5_), we determined the number of fish that passed in the first 5 min (F_5_) of each hour of day k. Then, we determined the number that passed in the first 10 min (F_10_) of each hour of the same day k, and so on until we determined the number of fish that passed for the entire 60 min (F_60_) of each hour of day k. Finally, we determined N_5_ (which is the sum of all F_5_ of day k or, in other words, the number of fish that passed in the first 5 min of all hours of day k), N_10_,… and N_60_. N_60_ is equal to the total number of fish that passed on day k, *i*.*e*., the true daily run size (D). We excluded days with D = 0 from the analyses. In addition to the 1 h sample interval (SI), we repeated the entire procedure described in this paragraph for SI = 2, 3, 4, 6 and 12 h.

### Data analysis

For SI = 1 h, we used simple linear regression to determine for each species and SU the regression coefficient (*b*), intercept (*a*) and coefficient of determination (*r*^*2*^) for the equation

$$D=a+bE_{su}$$ (1)

where E_su_ represents the estimate of D. We calculated E_su_ for every SU with the general equation

$$E_{su}=60N_{su}/SU$$ (2)

Thus, we calculated E_5_, which is the estimate of D for SU_5_, using the equation

$$E_{5}=60N_{5}/5$$ (3)

and E_10_ with

$$E_{10}=60N_{10}/10$$ (4)

and so on until E_60_ with the equation

$$E_{60}=60N_{60}/60$$ (5)

We also determined *b*, *a* and *r*^2^ for SI = 2, 3, 4, 6 and 12 h. For SI > 1, we estimated E_su_ using the same equations as before, but included SI as in

$$E_{su}=60N_{su}SI/SU$$ (6)

We determined *b*, *a* and *r*^*2*^ for each of the seven most abundant species in the counts, with all other species being grouped in the category ‘Others’.

When *b* and *r*^*2*^ are equal to 1.00, the sample estimates D with maximum accuracy. In the case of *b* = 1.00, any increase in E_su_ results in an increase of the same amount in D. As *b* deviates from 1.00, accuracy decreases. For example, when *b* = 0.50, the increase of one fish in the sample implies an increase of 0.50 fish in D. That is, the sample underestimates D. On the other hand, if *b* = 1.50, the increase in D would be 1.50 fish for each increase of one fish in E_su_, and the sample overestimates D. Regarding *r*^*2*^, the closer to 1.00 the better since there is no error in estimating D from E_su_ when *r*^*2*^ = 1.00. As *r*^*2*^ moves away from 1.00, the error increases and E_su_ becomes a less and less precise estimator of D.

We analyzed estimates of D considering three arbitrary classes of accuracy: high (0.95 ≤ *b* ≤ 1.05), medium (0.90 ≤ *b* ≤ 1.10, excluding the high accuracy range) and low (0.85 ≤ *b* ≤ 1.15, excluding the medium accuracy ranges). For the regression equations with *b* classified in one of the three accuracy categories, we determined whether *a* ≠ 0 by t-test at the significance level of 0.05.

For each species, we determined the daily sampling duration (DSD), defined as the minimum counting time per day (in minutes) required to estimate D. We determined DSD using the equation

DSD=24SU/SI (7)

Since the DSD of a given species can vary among accuracy classes, we determined DSD for each class. For a given accuracy, we used the lowest SI and SU with *r*^*2*^ ≥ 0.90 to calculate the DSD, from which the accuracy of estimating D was the same for all other SU values above the SU used in the equation.

For SI = 1 h, we classified the relationship between *b* and SU among four types: Type 1—*b* was relatively constant and close to 1.00 for all values of SU; Type 2—*b* showed a tendency to increase/decrease gradually with increasing SU until reaching 1.00; Type 3—*b* varied erratically; and Type 4—*b* was predominantly greater than 1.00.

Also for SI = 1 h, we determined the temporal pattern of fish passage through the fishway (more uniform or more aggregated) for each species based on the time interval between two consecutive passages. Thus, for each hour in which at least two fish passed through the fishway, we established the minute of passage for each individual from which we subtracted the minute of passage of the preceding fish. For the first fish to pass in an hour or when only one fish passed in an hour, the subtraction was done with zero. The time interval between two consecutive passages in the same minute was zero. With the results of the subtractions for each hour, we calculated the average time interval between passes. We then obtained the time passage deviation (TPD) determined by the average time interval between passages of each hour minus the time interval if all the fish of that hour had passed evenly distributed throughout the hour. We obtained this time interval by dividing 60 min by the number of fish that passed throughout the hour plus one fish. We added one more fish to match the minute of passage of the first fish of the hour with the time interval between the passage of the last fish and the end of the hour. The closer TPD is to zero, the more evenly distributed the fish passage is for the hour.

For each species and SI = 1, we evaluated the influence of fish abundance on b-SU relationship type, TPD and DSD. We made this assessment using four abundance classes. For the most abundant class, we used the number of individuals obtained in the images of the 46 days with fish counts. For the other three abundance classes, we reduced the number of days sampled to reduce the abundance. To do this, we randomly drew days stratified by lunar cycle. All lunar cycles but one had 4 days with fish counting. We excluded from the drawing the lunar cycle that did not have 4 days. We raffled two days per lunar cycle to reduce the number of days to 22, one day per lunar cycle to reduce to 11 days, and one day for every two consecutive lunar cycles to reduce to 6 days. For each abundance class, we determined b-SU relationship type, TPD and DSD (only for the high accuracy class) as previously described. We compared b-SU relationship type among the abundance classes and determined the linear regression of TPD and DSD using the log of the number of fish in each abundance class. We tested whether the linear regression coefficient was significant using the t-test at the significance level of 0.05.

We carried out all data processing and analysis in SAS and plots in Excel and Statistical [16–18].

## Results

**Fig 2:**
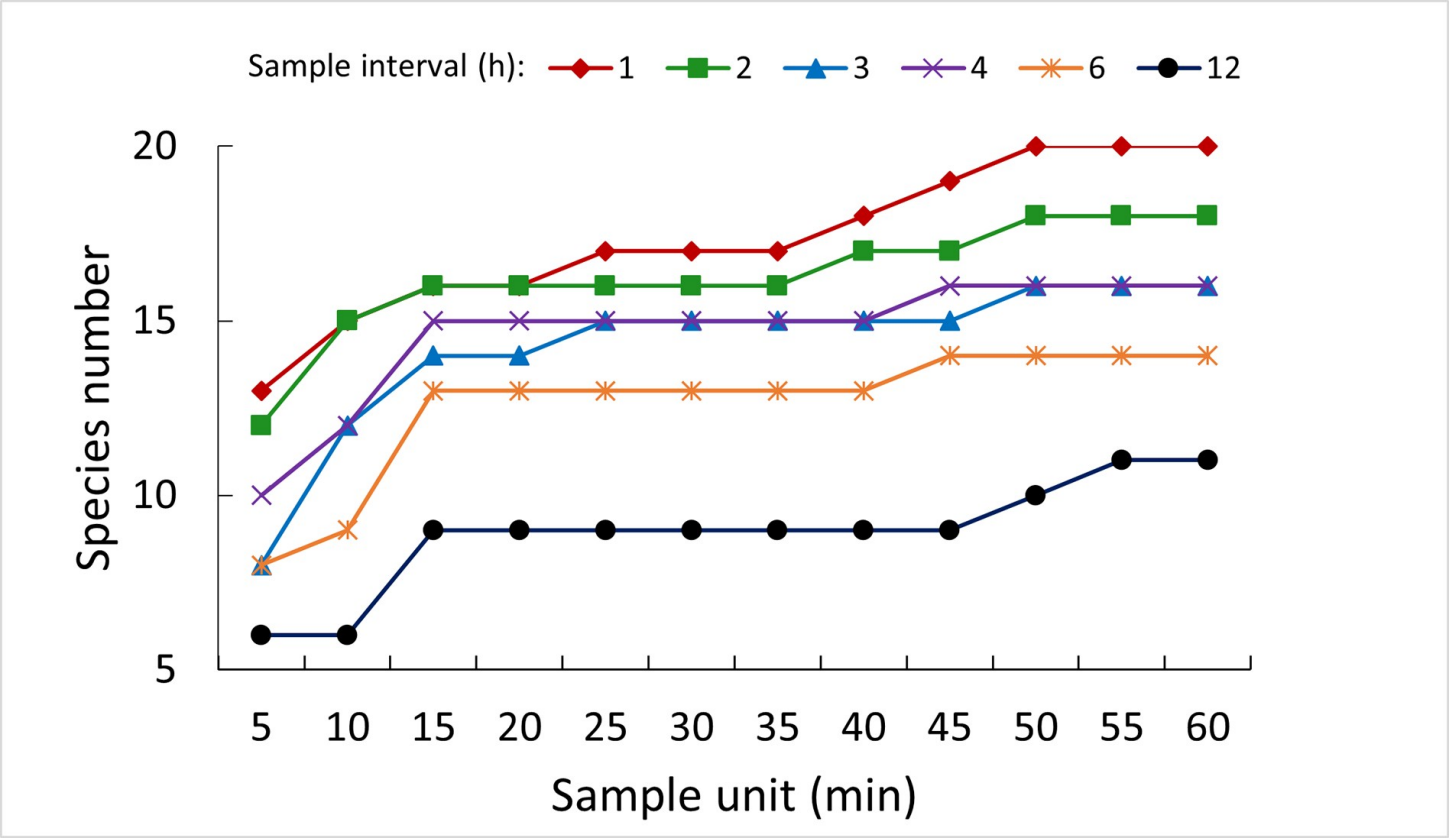
Number of fish species by sample unit and sample interval. Number of fish species determined by systematic sampling of the passage of fish at the Igarapava Fish Ladder.

We counted a total of 12,097 fish of at least 20 species in the images (S1 Table). The seven dominant species in abundance rank were *Leporinus octofasciatus*, *Pimelodus maculatus*, *Leporinus friderici*, *Prochilodus lineatus*, *Piabarchus stramineus*, *Schizodon nasutus*, and *Salminus hilarii*. These species represented 98.2% of all the individuals counted. The remaining 12 species plus *Cichla* spp., grouped in the ‘Others’ category, accounted for 265 individuals. The number of species increased with increasing SU for all SI values and reached 20 only when SI = 1 h and SU ≥ 50 min (Fig 2).

**Fig 3:**
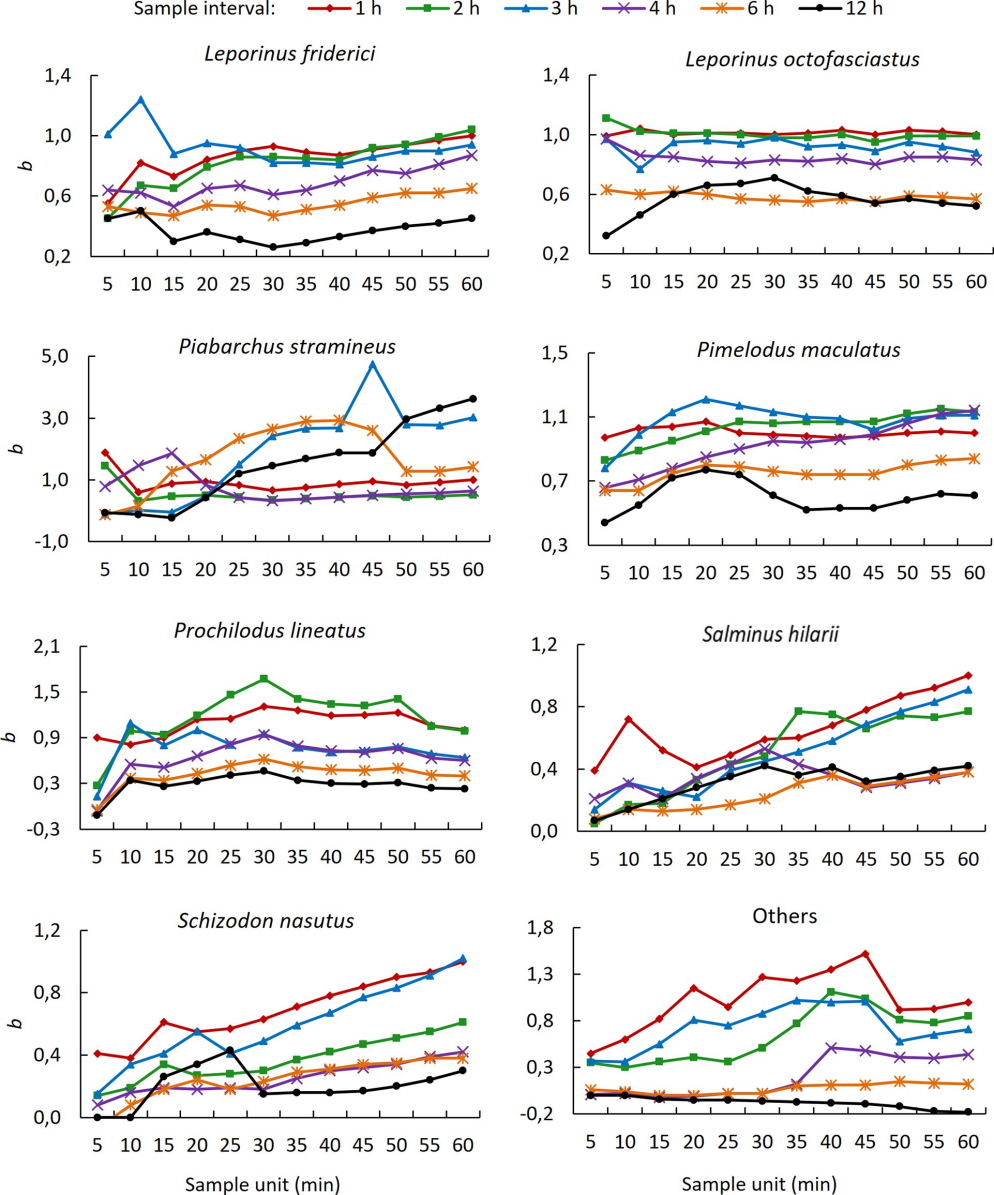
Relationship between the linear regression coefficient (*b*) and sample unit. Sample unit and *b* for six sample intervals for the seven most abundant fish species and species of low abundance grouped as ‘Others’ in the Igaparava Fish Ladder.

For SI = 1 h, *b* increased with increasing SU for most species, the exceptions being *P*. *stramineus* and *P*. *lineatus* (Fig 3). When SI ≤ 3 h, *b* values between 0.95 and 1.05 were frequent, mainly for the two most abundant species (Fig 3). Values of *b* between 0.95 and 1.05 for SI = 4 h were rare and occurred only for *L*. *octofasciatus* and *P*. *maculatus*. For all species, including ‘Others’, SI = 6 h and SI = 12 h resulted mostly in *b* values distant from 1.00 (i.e., ≤ 0.85 or ≥ 1.15).

**Fig 4:**
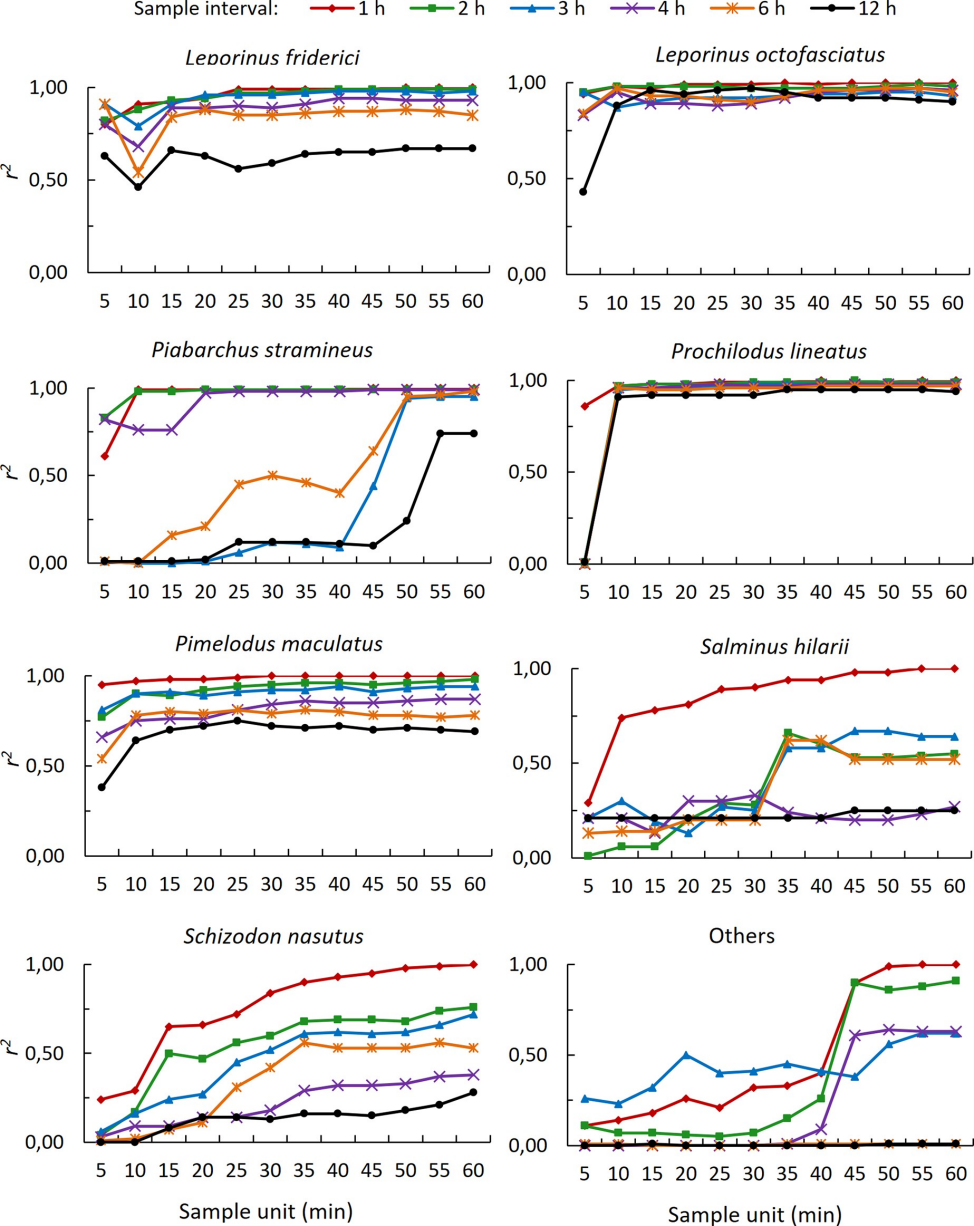
Relationship between the coefficient of determination (*r*^*2*^) and sample unit. Sample unit and *r*^*2*^ for six sample intervals for the seven most abundant fish species and species of low abundance grouped as ‘Others’ in the Igaparava Fish Ladder.

Values of *r*^*2*^ tended toward 1.00 with increasing SU and a reduction in SI for all species, as well as for ‘Others’ (Fig 4). For the five most abundant species, when SI was lower, high *r*^*2*^ (≥ 0.90) occurred consistently from low SU. On the other hand, for the two less abundant species (*S*. *nasutus* and *S*. *hilarii*), high *r*^*2*^ occurred only with the highest values of SU associated with SI = 1.

Values of DSD varied among species and, for almost all of them, decreased with the reduction of accuracy in the estimate of D (Table 1). For *P*. *maculatus*, the DSD of the high class of accuracy in the estimate of D was 600 min. It was calculated with SI = 1 h and SU = 25 min, since the accuracy of estimating D was high for all SU values greater than 25 min when SI = 1 h. The 600 min DSD represents 41.7% of the 1,440 min of a day. The DSD of the other most abundant species in the high accuracy class ranged from 120 to 1,440 min (Table 1), that is, 8.3 to 100.0% of the minutes of the day. The smallest DSD values, those ≤ 660 min, were for species with a *b*-SU relationship of Type 1 (*L*. *octofasciatus*, *P*. *maculatus*), Type 2 (*L*. *friderici*) and Type 4 (*P*. *lineatus*) while the largest DSD values (= 1,440 min) occurred with those of Type 2 (*S*. *nasutus*) and Type 3 (*P*. *stramineus*, *S*. *hilarii* and ‘Others’). The decrease in accuracy in the estimate of D from high to medium reduced the DSD for all species, except *L*. *octofasciatus* and *P*. *lineatus*, two fishes that already had low DSD even at high accuracy. The reduction in accuracy from high to low failed to reduce the DSD for only *P*. *lineatus*. The lowest DSD was for 60 min (i.e., 4.2% of the day) and occurred with low accuracy for *L*. *octofasciatus*.

**Table 1:** Metrics for the daily run-size estimation method.

Species	Class of accuracy in the estimate of D														
	High (0.95 ≤ *b* ≤ 1.05)					Medium (0.90 ≤ *b* ≤ 1.10, excluding the high accuracy range)					Low (0.85 ≤ *b* ≤ 1.15, excluding the medium accuracy ranges)				
	SI (h)	SU (min)	DSD (min)	*r*^*2*^	*a*	SI (h)	SU (min)	DSD (min)	*r*^*2*^	*a*	SI (h)	SU (min)	DSD (min)	*r*^*2*^	*a*
*L*. *friderici*	2	55	660	0.99	2.5	3	50	400	0.98	2.8	3	45	360	0.98	3.1
*L*. *octofasciatus*	2	10	120	0.98	11.8	2	10	120	0.98	11.8	2	5	60	0.95	6.9
*P*. *lineatus*	2	55	660	0.99	1.9	2	55	660	0.97	1.9	2	55	660	0.99	1.9
*P*. *maculatus*	1	25	600	0.99	0.1	1	5	120	0.95	1.8	1	5	120	0.95	1.8
*P*. *stramineus*	1	60	1440	1.00	0.0	1	55	1320	1.00	0.9	1	55	1320	1.00	0.9
*S*. *hilarii*	1	60	1440	1.00	0.0	1	55	1320	1.00	0.1	1	50	1200	0.98	0.2
*S*. *nasutus*	1	60	1440	1.00	0.0	1	50	1200	0.90	0.3	1	50	1200	0.98	0.3
Others	1	60	1440	1.00	0.0	1	50	1200	0.99	0.3	1	50	1200	0.99	0.3

Sample interval (SI), sample unit (SU), daily sampling duration (DSD), coefficient of determination (*r*^*2*^) and intercept (*a*) of the linear regression to estimate daily run size (D) for three classes of accuracy of D determined for the seven most abundant fish species and species of low abundance grouped as ‘Others’ in the Igarapava Fish Ladder. For species with more than one DSD value per accuracy class, only the metrics for the DSD with the highest *r*^*2*^ are shown in the table. All values of *a* are not significantly different from zero according to t-test.

The two most abundant species (*L*. *octofasciatus* and *P*. *maculatus*) presented the Type 1 *b*-SU relationship, while the third (*L*. *friderici*) and seventh (*S*. *nasutus*) showed Type 2, the fifth (*P*. *stramineus*), sixth (*S*. *hilarii*) and eighth (*‘*Others’) exhibited Type 3 and the fourth (*P*. *lineatus*) presented Type 4 (Table 2).

**Table 2:** Number of fish and *b*-SU relationship type per number of sampling days.

Species	Number of sampling days							
	46		22		11		6	
	Number of fish	*b*-SU relationship type	Number of fish	*b*-SU relationship type	Number of fish	*b*-SU relationship type	Number of fish	*b*-SU relationship type
*L*. *octofasciatus*	4.756	1	1.703	2	1.189	2	234	3
*P*. *maculatus*	4.250	1	2.159	2	1.256	2	336	2
*L*. *friderici*	1.238	2	653	2	143	3	110	3
*P*. *lineatus*	645	4	232	2	168	2	60	3
*P*. *stramineus*	615	3	550	3	24	2	30	2
*S*. *nasutus*	241	2	106	2	20	2	34	3
*S*. *hilarii*	87	3	59	2	38	3	1	..
Others	265	3	151	3	47	2	12	3

Number of fish counted and *b*-SU relationship type per number of sampling days for the seven most abundant fish species and species of low abundance grouped as ‘Others’ in the Igarapava Fish Ladder.

**Fig 5:**
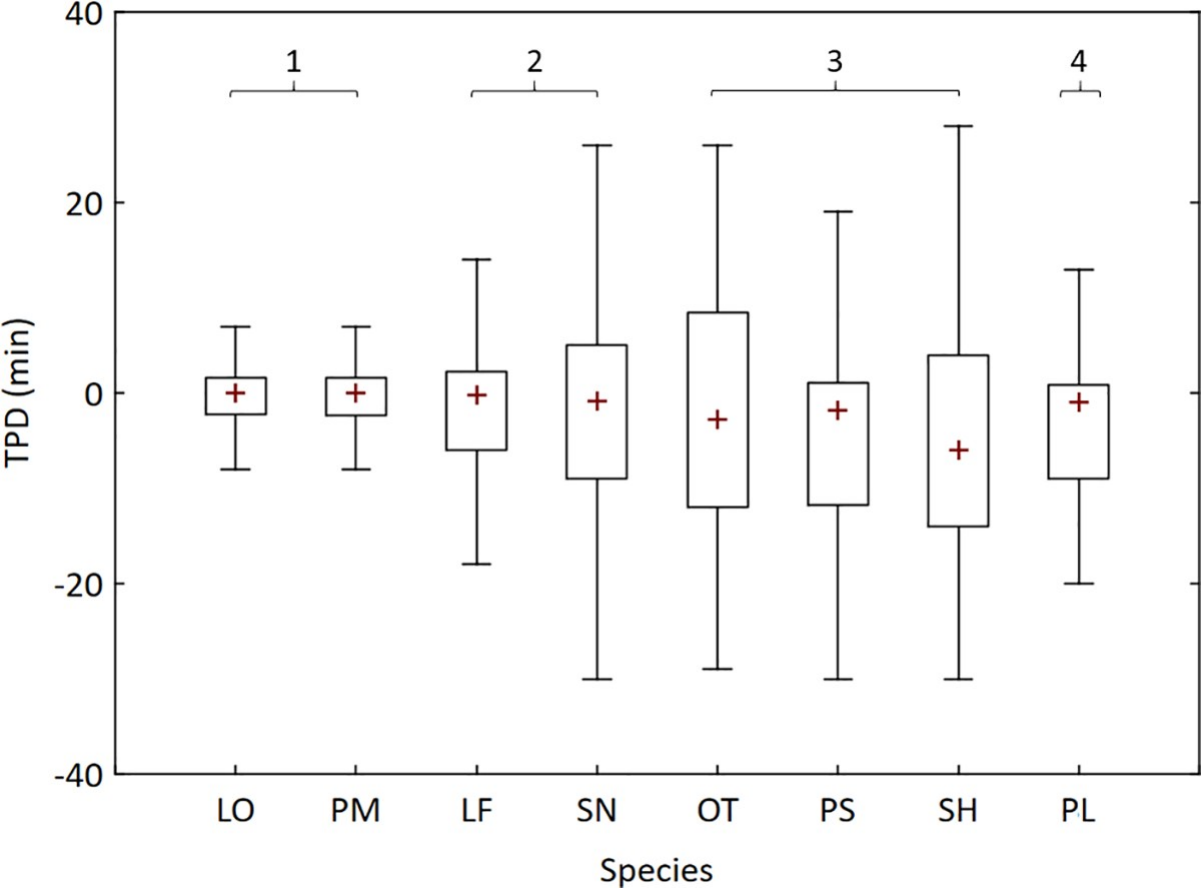
Boxplot of Time Passage Deviation (TPD) per species. TPD for the seven most abundant fish species and species of low abundance grouped as ‘Others’ in the Igaparava Fish Ladder. The closer TPD is to zero the more uniformly distributed was the passage throughout the hour (LO: *L*. *octofasciatus*, PM: *P*. *maculatus*, LF: *L*. *friderici*, SN: *S*. *nasutus*, OT: ‘Others’, PS: *P*. *stramineus*, SH: *S*. *hilarii* and PL: *P*. *lineatus*). Numbers above boxplots indicate the *b*-SU relationship type. Boxplot shows median, interquartile interval and non-outlier range.

The medians for TPD were similar among all species, but both the interquartile intervals and non-outlier ranges differed (Fig 5). The interquartile intervals and non-outlier ranges of TPD were smaller for the two species with the Type 1 *b*-SU relationship, intermediary for *L*. *friderici* (Type 2) and *P*. *lineatus* (Type 4) and larger for the remaining species (Fig 5). The interquartile interval was approximately 3.9 min for the two species with the Type 1 *b*-SU relationship, 9.0 min for *L*. *friderici* and *P*. *lineatus* and ranged 12.9–20.5 min for the remain species.

Reduction in the number of sampling days reduced the abundance of fish except for *S*. *nasutus* when the number of sampling days reduced from 11 to 6 (Table 2). Reduction in abundance changed *b*-SU relationship type, at least once for each species, including for ‘Others’. It changed eight times to a higher-level type in six species plus ‘Others’ (e.g., *L*. *octofasciatus* and *P*. *maculatus* when the number of sampling days reduced from 46 to 22) and to a lower-level type four times in three species plus ‘Others’ (e.g., *P*. *lineatus* and *S*. *nasutus* when the number of sampling days reduced from 46 to 22).

**Fig 6:**
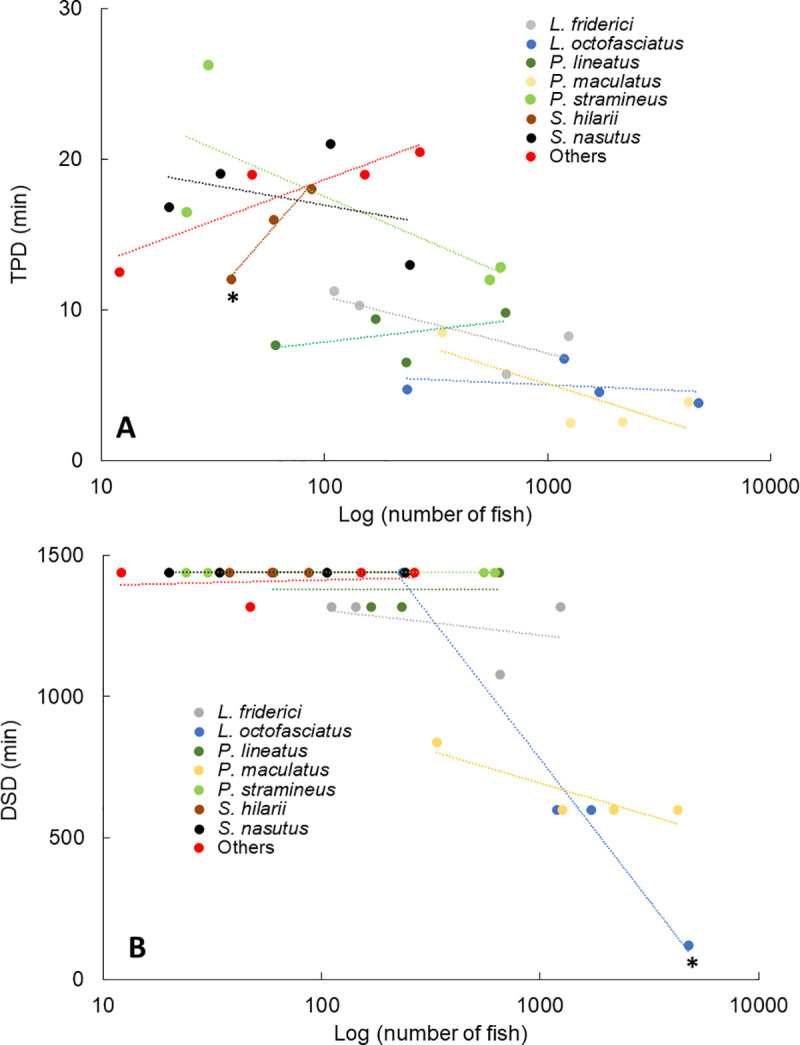
Influence of fish abundance on the interquartile interval of Time Passage Deviation (TPD) and Daily Sampling Duration (DSD). Regressions showing the influence of fish abundance on TPD (A) and DSD (B) for the seven most abundant fish species and species of low abundance grouped as ‘Others’ in the Igaparava Fish Ladder. Asterisk indicates linear regressions with a significant slope.

Independent of species, the interquartile interval of TPD increased with reduced fish abundance (Fig 6A). Conversely, when considering species, abundance influenced the interquartile interval of TPD only for *S*. *hilarii* (linear regression: *b* = 8.91; *P* = 0.01; Fig 6A). Similarly, fish abundance affected DSD only for *L*. *octofasciatus* (*b* = -41.8; *P* < 0.01; Fig 6B), but DSD was relatively low (≤ 600 min) for this species except when abundance was the lowest.

## Discussion

Our results indicate that it is possible to estimate D for species with a more uniform temporal pattern of passage using the DARSE method, with a reduction of up to 96% in the time required for sampling. Reduction in sampling time was achieved for some species by counting the fish that pass in a fraction of an hour for all hours of the day, while for others it was sufficient to count for a fraction of an hour every 2 h, but rarely 3 h. For species with more aggregated patterns of fish passage, sampling for a fraction of an hour can be done only with reduced accuracy of the D estimate. The temporal pattern of fish passage likely affects the accuracy of estimating D more than fish abundance. The type of *b*-SU relationship, which is not related to fish abundance, affects the better combination of SU and SI for estimating D. This combination varies among species and may need to be determined periodically.

Our interest was to develop a method to estimate daily run size of fish, not seasonal or annual run sizes as done in the retrospective studies of Jessop Harvie [4], Davies et al. [6] and McCormick [9]. Using the DARSE method to estimate seasonal or annual run size would require daily counts or counts on a certain number of days sampled throughout the season or year. However, run size can be sufficiently variable over a season or year to impose difficulties in defining the most appropriate sampling scheme for sampling days [4]. It has been suggested that stratified sampling by abundance should be used instead of simple random sampling when sampling days to estimate seasonal or annual run size because it is more accurate [9]. In fishways with high variation in daily run size determined by environmental factors, such as the case with IFL [14], stratifying by the environmental factor(s) with the greatest effect size on daily run size may be more appropriate than stratifying by abundance, but this hypothesis needs to be evaluated. Additionally, stratifying by that(those) environmental factor(s) is generally easier to implement than stratifying by abundance because (i) no prior information on abundance for the period of estimation is required and (ii) measuring environmental factor(s) is simpler than quantifying abundance.

The values obtained for *b* and *r*^*2*^ by Eq (1) indicate that it is possible to reduce DSD and estimate D with high accuracy for the species with a more uniform temporal pattern of passage. However, it was not possible to reduce DSD for species with a more aggregated temporal pattern of passage. For these species, all fish that pass in an hour need to be counted if high accuracy is desired. Reduction in accuracy from high to medium or low did little to reduce the DSD for them. The retrospective studies of Jessop & Harvie [4], Davies et al. [6] and McCormick et al. [9] also concluded that it is possible to reduce sampling effort and estimate run size with known accuracy. According to McCormick et al. [9], sampling more than 8 h per day, which represents a DSD of 480 min, increased accuracy very little. Our results showed that a DSD of up to 480 min was sufficient to accurately estimate D for a few species but DSD greater than 480 min provided higher accuracy for most species.

Of the four types of *b*-SU relationships, only Type 1 allows estimating D with high accuracy even for low SU, because *b* is relatively constant and close to 1.00 for most SU values. For the three other *b*-SU relationships, estimating D from a given SU is more restricted. In Type 2 and Type 4 *b*-SU relationships, for example, it was possible to estimate D with high accuracy only for species that presented a more uniform temporal pattern of passage, but with higher SU. For the Type 3 *b*-SU relationship, estimation of D was possible but only with reduced accuracy. The reduction of fish abundance by reducing the number of sampled days changed the type of *b*-SU relationship for all taxa analyzed. The direction of the change in type, if to a lower- or higher level, was not related to abundance.

The relatively constant values of *b* close to 1.00 for the Type 1 *b*-SU relationship indicate a more uniform temporal pattern of passage of fish throughout the hour. This is supported by the smaller interquartile intervals of TPD of the species with the Type 1 *b*-SU relationship compared to species with the other types. A smaller interquartile interval for TPD means a greater frequency of hours with TPD closer to zero compare to a larger interquartile interval. The closer that TPD is to zero, the more uniform is the fish passage throughout the hour. For a hypothetical fish species for which the same number of individuals passes at equal time intervals throughout the hour, *b* will be exactly 1.00 independent of SU, D and the number of fish that pass each hour. The greater the variation in both the number of individuals and the time interval between passages, the further *b* will be from 1.00.

The erratic variation of *b* for the Type 3 *b*-SU relationship had two causes: first, the inaccurate estimation of D for the least abundant species and, second, the passage of schools with proportionally high numbers of fish at irregular intervals. *Salminus hilarii* exemplifies the first cause. The species was recorded on only 20 days when D ranged 1–20, and on 10 of those days, D was less than 3. For such low D values, the probability of E_su_ being equal to zero is high, particularly for lower SU. Therefore, low abundance associated with many days with D near zero caused the inaccurate estimation of D for *S*. *hilarii*. The second cause is exemplified by *P*. *stramineus*, whose *b*-SU relationship changed to Type 2 after we excluded 10 schools that accounted for 64% of the individuals counted and passed at irregular intervals within just 8 h of the same day. The aggregate passage of fish in a single day also negatively affects the estimation of D for species with the Type 4 *b*-SU relationship. For *P*. *lineatus*, a fish that forms large schools [19, 20], *b* was predominantly > 1.00 due to the passage of 60% of all counted fish in a single day. The elimination of that day changed the *b*-SU relationship for *P*. *lineatus* from Type 4 to Type 2.

The temporal pattern of fish passage likely affects the accuracy of estimating D more so than does fish abundance. We were able to estimate D with high accuracy by sampling a fraction of an hour for the three most abundant species (all showed a more uniform temporal pattern of passage), even when we reduced their abundance. The exception was *L*. *octofasciatus*, and only when the number of sampled days was reduced from 46 to 6 days. Moreover, the temporal pattern of passage for this species was not affected by the reduction in abundance. Conversely, we could not estimate D with high accuracy for any of the other species, including “Others”, with a more aggregated temporal pattern of passage most likely because of their higher interquartile interval of TPD. Their lower abundance seemed not to be the reason we could not estimate D with high accuracy because lower abundance did not affect the capacity of estimating D with high accuracy for the species with a more uniform temporal pattern of passage. For fishes with a more aggregated temporal pattern of passage, estimating D from a sample of a fraction of an hour is only possible with reduced accuracy and with little savings in DSD.

The most suitable SU and SI for estimating D varied among the studied species. Thus, the choice of SU and SI should be made for each target species. The study McCormick et al. [9] indicated that differences in daily and seasonal run timing may not allow one sampling design to be optimal for all species that pass a fishway. One of the advantages of the DARSE method is that its sampling design is the same for all species, differing only in the SU and SI. A SI value greater than 4 h is not recommended since accuracy in the estimation of D was less than low for all the studied fishes. For most of them, *b* closer to 1.00 was obtained for SI ≤ 3 h. This likely occurred because distributing the DSD throughout the day results in greater accuracy than sampling the same amount of time but grouped [6, 9–11, 21].

It might be necessary to determine SU and SI periodically (e.g., every year or so) because they may vary with time, but more studies on that are needed. We could not evaluate annual variation of SU and SI because we had only one year of data. Determining SU and SI annually for a long time series may provide valuable data for determining if they indeed vary in time and what causes such variation. Additionally, we found that less abundant species were associated with a more aggregated temporal pattern of passage, while more abundant species had a more uniform temporal pattern of passage. Reducing the abundance of the more abundant species did not affect their temporal pattern of passage. This suggests that the relationship we found between abundance and temporal pattern of passage could be a spurious one, but more investigation on the actual relationship is also necessary and essential because abundance may vary for the same species in different basins and even in different fishways in the same basin [22, 23].

Choosing SU is particularly critical for species with a more aggregated temporal pattern of passage. A fixed SU has been used to sample fishways to estimate run size (e.g., [8, 11, 24]). Fixed SU was also adopted in the retrospective sampling studies of Jessop Harvie [4] and Davies et al. [6]. Instead of defining a fixed SU *a priori*, we used retrospective sampling to define how long the SU should be. Defining SU *a posteriori* seems a better approach for fishes with a more aggregated temporal pattern of passage because a SU near or equal to 60 min was necessary to determine their daily run size.

Since determining daily run size by counting all fish can be overly laborious, reducing sampling effort optimizes the use of resources. The DARSE method allows for considerable reduction in sampling effort for the species with a more uniform temporal pattern of passage. It also makes it possible to assess how much of the sampling effort can be reduced to estimate daily run size with the desired accuracy. The reduction in sampling effort must, however, be in line with the objectives of the fishway. One of the frequent main goals of upstream fishways is allowing access to spawning sites [1, 11, 25, 26], but there can be other purposes [27, 28]. The relevance of identifying passages of a few individuals of certain species, like the non-migrant and endangered *Myloplus tiete* recorded in our counts only with SU = 50 min and SI = 1, may depend on the use of the fishway as a tool for management, and always considering the life cycle and needs of each species [29, 30].

The DARSE method reduced the sampling effort needed to estimate daily run size through the IFL, but its use seems to depend on the temporal pattern of fish passage of the target species. The proposed methodology needs to be tested for other species, other fishways and/or over longer time series to assess whether the same temporal pattern of passage is obtained. Certainly, individual-based model [31] is a powerful tool for testing hypotheses about the influence of temporal patterns of passage on the parameters of the DARSE method, like SU, SI, TPD and the *b*-SU relationship. Step-by-step instructions for using this method are provided in S1 Appendix.

## Supporting information

S1 Appendix Step-by-step instructions for the use of the DARSE method. (DOCX)

S1 Table Number of individuals counted per species in video-images of different sample units on taken during 46 days at the Igarapava Fish Ladder. (DOCX)

S1 Dataset Data set for fish passage at the Igarapava Fish Ladder. (XLSX)
