# Supplementary material for: Less effort but equal result: Introducing the daily run-size estimation method for quantifying fish passage in fishways
Source: PLoS One. 2021 May 26;16(5):e0252183. doi: 10.1371/journal.pone.0252183 (PMC8153445; doi:10.1371/journal.pone.0252183)
Supplement: S1 Appendix — (DOCX) [file pone.0252183.s001.docx]

# STEP-BY-STEP INSTRUCTIONS FOR THE USE OF THE DAILY RUN SIZE ESTIMATION METHOD

1. Count the number of fish of the target species that pass through the fishway every 5 min of every day of a certain period (e.g., season or year) or samples of days. See [1] to define the type of sampling to sample days.
2. For the first day of counting, determine the number of fish that pass in the first 5 min of each hour (F_5_). These first five minutes correspond to the sample unit (SU) equal to 5 min. Repeat the process for SU = 10 min to obtain F_10_, then for SU = 15 to obtain F_15_ and so on until F_60_, as shown in Tab. 1.
3. Also, for the first day of counting, sum all the values of F_5_ to determine N_5_, all of F_10_ to obtain N_10_ and so on until N_60_ (Tab. 1).
4. Estimate the number of fish that passed during the first day (E) for SU = 5 by equation E_5_ = 60N_5_/SU (Tab. 1).
5. Repeat step 4 for SU = 10, SU = 15, ..., until SU = 60. When SU = 60, E = D (Tab. 1).
6. Repeat steps 2 thru 5 for all counting days.
7. With the values of E_5_ obtained for all counting days, perform a simple linear regression of D on E_5_ to determine *b* and *r^2^*.
8. Repeat step 7 for E_10_, E_15_, ..., until E_60_.
9. Repeat steps 2 thru 8 for sample intervals of 2, 3, 4, 6 and 12 h.
10. Repeat steps 1 thru 9 for the other target species, if any.

Table 1. Counts of *Leporinus octofasciatus* per sample unit and hour of the day at the Igarapava Fish Ladder on 13 Jan 2004.

| Hour | Counts of *Leporinus octofasciatus* per sample unit (min) | | | | | | | | | | | |
| --- | --- | --- | --- | --- | --- | --- | --- | --- | --- | --- | --- | --- |
|  | 5 | 10 | 15 | 20 | 25 | 30 | 35 | 40 | 45 | 50 | 55 | 60 |
| 0 | 0 | 0 | 0 | 0 | 0 | 0 | 0 | 0 | 0 | 0 | 0 | 0 |
| 1 | 0 | 0 | 0 | 0 | 0 | 0 | 0 | 0 | 0 | 0 | 0 | 0 |
| 2 | 0 | 0 | 0 | 0 | 0 | 0 | 0 | 0 | 0 | 0 | 0 | 0 |
| 3 | 0 | 0 | 0 | 0 | 0 | 0 | 0 | 0 | 0 | 0 | 0 | 0 |
| 4 | 0 | 0 | 0 | 0 | 0 | 0 | 0 | 0 | 7 | 16 | 20 | 22 |
| 5 | 6 | 8 | 12 | 12 | 17 | 21 | 32 | 37 | 42 | 45 | 49 | 50 |
| 6 | 0 | 1 | 4 | 9 | 13 | 17 | 18 | 21 | 28 | 34 | 34 | 34 |
| 7 | 0 | 0 | 0 | 0 | 0 | 0 | 0 | 0 | 0 | 0 | 0 | 0 |
| 8 | 0 | 0 | 1 | 1 | 1 | 1 | 1 | 1 | 1 | 2 | 2 | 2 |
| 9 | 0 | 0 | 0 | 0 | 0 | 0 | 0 | 0 | 0 | 0 | 0 | 0 |
| 10 | 0 | 0 | 0 | 0 | 0 | 0 | 2 | 4 | 4 | 4 | 4 | 4 |
| 11 | 0 | 0 | 0 | 0 | 0 | 0 | 0 | 0 | 0 | 0 | 0 | 1 |
| 12 | 0 | 0 | 0 | 1 | 1 | 1 | 1 | 4 | 4 | 4 | 4 | 5 |
| 13 | 0 | 0 | 3 | 3 | 3 | 3 | 3 | 3 | 3 | 3 | 8 | 9 |
| 14 | 0 | 2 | 2 | 6 | 7 | 7 | 9 | 11 | 12 | 13 | 13 | 13 |
| 15 | 4 | 11 | 12 | 14 | 20 | 20 | 21 | 21 | 21 | 21 | 21 | 21 |
| 16 | 0 | 0 | 3 | 4 | 8 | 11 | 11 | 11 | 11 | 12 | 14 | 16 |
| 17 | 0 | 1 | 2 | 5 | 5 | 6 | 6 | 6 | 7 | 12 | 12 | 13 |
| 18 | 0 | 0 | 0 | 0 | 0 | 0 | 0 | 0 | 0 | 0 | 0 | 0 |
| 19 | 0 | 0 | 0 | 0 | 0 | 0 | 0 | 0 | 0 | 0 | 0 | 0 |
| 20 | 0 | 0 | 0 | 0 | 0 | 0 | 0 | 0 | 0 | 0 | 0 | 0 |
| 21 | 0 | 0 | 0 | 0 | 0 | 0 | 0 | 0 | 0 | 0 | 0 | 0 |
| 22 | 0 | 0 | 0 | 0 | 0 | 0 | 0 | 0 | 0 | 0 | 0 | 0 |
| 23 | 0 | 0 | 0 | 0 | 0 | 0 | 0 | 0 | 0 | 0 | 0 | 0 |
| N | 10 | 23 | 39 | 55 | 75 | 87 | 104 | 119 | 140 | 166 | 181 | 190 |
| E | 120 | 138 | 156 | 165 | 180 | 174 | 178 | 179 | 187 | 199 | 197 | 190 |

**References**

1. McCormick JL, Jackson LS, Carr FM, Meyer DH. Evaluation of probabilistic sampling designs for estimating abundance of multiple species of migratory fish using video recordings at fishways. N Am J Fish Manag. 2015; 35:818–826.
