# Supplementary material for: Less effort but equal result: Introducing the daily run-size estimation method for quantifying fish passage in fishways
Source: PLoS One. 2021 May 26;16(5):e0252183. doi: 10.1371/journal.pone.0252183 (PMC8153445; doi:10.1371/journal.pone.0252183)
Supplement: S1 Table — (DOCX) [file pone.0252183.s002.docx]

**S1 Table.** **Number of individuals counted per species in video-images of different sample units on 46 days at the Igarapava Fish Ladder.**

| Species | Sample unit (min) | | | | | | | | | | | |  |
| --- | --- | --- | --- | --- | --- | --- | --- | --- | --- | --- | --- | --- | --- |
|  | 5 | 10 | 15 | 20 | 25 | 30 | 35 | 40 | 45 | 50 | 55 | 60 | |
| *Leporinus octofasciatus^m^* | 371 | 753 | 1,191 | 1,563 | 1,968 | 2,382 | 2,754 | 3,117 | 3,548 | 3,933 | 4,327 | 4,756 | |
| *Pimelodus maculatus^m^* | 359 | 722 | 1,032 | 1,344 | 1,761 | 2,130 | 2,463 | 2,856 | 3,177 | 3,542 | 3,894 | 4,250 | |
| *Leporinus friderici^m^* | 116 | 208 | 354 | 435 | 558 | 648 | 785 | 901 | 984 | 1,081 | 1,154 | 1,238 | |
| *Prochilodus lineatus^m^* | 57 | 111 | 165 | 189 | 237 | 254 | 306 | 380 | 433 | 470 | 570 | 645 | |
| *Piabarchus stramineus* | 33 | 159 | 173 | 212 | 287 | 414 | 426 | 435 | 480 | 576 | 602 | 615 | |
| *Schizodon nasutus^m^* | 16 | 32 | 62 | 90 | 115 | 144 | 162 | 178 | 192 | 214 | 231 | 241 | |
| *Salminus hilarii^m^* | 6 | 13 | 26 | 43 | 50 | 53 | 65 | 68 | 77 | 79 | 85 | 87 | |
| *Astyanax lacustris* | 2 | 5 | 9 | 12 | 18 | 18 | 22 | 24 | 47 | 76 | 83 | 83 | |
| *Cichla* spp. | 3 | 7 | 13 | 14 | 17 | 27 | 35 | 40 | 41 | 46 | 51 | 54 | |
| *Metynnis maculatus* | 3 | 6 | 7 | 8 | 9 | 11 | 11 | 12 | 19 | 41 | 45 | 49 | |
| *Megalancistrus parananus^m^* | 3 | 8 | 10 | 11 | 15 | 17 | 18 | 21 | 23 | 27 | 30 | 32 | |
| *Clarias gariepinus* | 0 | 3 | 5 | 5 | 8 | 8 | 10 | 11 | 13 | 16 | 19 | 20 | |
| *Brycon orbignyanus^m*^* | 0 | 0 | 3 | 5 | 6 | 6 | 6 | 7 | 8 | 8 | 8 | 8 | |
| *Piaractus mesopotamicus^m^* | 0 | 1 | 2 | 3 | 3 | 4 | 4 | 6 | 6 | 6 | 6 | 6 | |
| *Myloplus tiete** | 0 | 0 | 0 | 0 | 0 | 0 | 0 | 0 | 0 | 4 | 5 | 5 | |
| *Galeocharax knerii* | 1 | 1 | 2 | 3 | 3 | 3 | 3 | 3 | 3 | 3 | 4 | 4 | |
| *Acestrorhynchus lacustris* | 1 | 1 | 1 | 1 | 1 | 1 | 1 | 1 | 1 | 1 | 1 | 1 | |
| *Brycon nattereri^m^* | 0 | 0 | 0 | 0 | 1 | 1 | 1 | 1 | 1 | 1 | 1 | 1 | |
| *Loricaria lentiginosa* | 0 | 0 | 0 | 0 | 0 | 0 | 0 | 0 | 1 | 1 | 1 | 1 | |
| *Salminus brasiliensis^m^* | 0 | 0 | 0 | 0 | 0 | 0 | 0 | 1 | 1 | 1 | 1 | 1 | |
| Number of individuals | 971 | 2,030 | 3,055 | 3,938 | 5,057 | 6,121 | 7,072 | 8,062 | 9,055 | 10,126 | 11,118 | 12,097 | |
| Number of species | 13 | 15 | 16 | 16 | 17 | 17 | 17 | 18 | 19 | 20 | 20 | 20 | |

^m^ Migratory species [1]; * Endangered species [2]

**References**

1. Agostinho AA, Gomes LC, Suzuki HI, Júlio Jr HF. Migratory fishes of the Upper Paraná River Basin, Brazil. In: Carolsfeld J, Harvey B, Ross C, Baer A, editors. Biology, Fisheries and Conservation Status. Canada: The International Bank for Reconstruction and Development / The World Bank; 2003. p.19-98.

2. Instituto Chico Mendes de Conservação da Biodiversidade. Executive Summary. Brazil Red Book of Threatened Species of Fauna. 2016. 76p.
